# CRISPR/Cas9 generated knockout mice lacking phenylalanine hydroxylase protein as a novel preclinical model for human phenylketonuria

**DOI:** 10.1038/s41598-021-86663-8

**Published:** 2021-03-31

**Authors:** Kuldeep Singh, Cathleen S. Cornell, Robert Jackson, Mostafa Kabiri, Michael Phipps, Mitul Desai, Robert Fogle, Xiaoyou Ying, Gulbenk Anarat-Cappillino, Sarah Geller, Jennifer Johnson, Errin Roberts, Katie Malley, Tim Devlin, Matthew DeRiso, Patricia Berthelette, Yao V. Zhang, Susan Ryan, Srinivas Rao, Beth L. Thurberg, Dinesh S. Bangari, Sirkka Kyostio-Moore

**Affiliations:** 1 Global Discovery Pathology, Translational In-Vivo Models Research Platform, Sanofi, 5 The Mountain Road, Framingham, MA 01701 USA; 2 Genomic Medicine Unit, Sanofi, 49 New York Avenue, Framingham, MA 01701 USA; 3 Transgenic Model and Technology, Translational In-Vivo Models Research Platform, Sanofi, 5 The Mountain Road, Framingham, MA 01701 USA; 4 Transgenic Model and Technology, Translational In-Vivo Research Platform, Industrie Park Hoechst, Sanofi, Frankfurt, Germany; 5 Global Bioimaging, Translational In-Vivo Models Research Platform, Sanofi, Framingham, MA 01701 USA; 6 Pre-Development Sciences NA, Analytical R&D, Sanofi, Framingham, MA 01701 USA; 7 Translational In-Vivo Models Research Platform, Sanofi, 49 New York Avenue, Framingham, MA 01701 USA; 8 Present Address: WuXi AppTec Inc., 8th Floor, 55 Cambridge Parkway, Cambridge, MA 02142 USA

**Keywords:** Diseases, Pathogenesis, Experimental models of disease, Preclinical research, Translational research

## Abstract

Phenylketonuria (PKU) is an autosomal recessive inborn error of l-phenylalanine (Phe) metabolism. It is caused by a partial or complete deficiency of the enzyme phenylalanine hydroxylase (PAH), which is necessary for conversion of Phe to tyrosine (Tyr). This metabolic error results in buildup of Phe and reduction of Tyr concentration in blood and in the brain, leading to neurological disease and intellectual deficits. Patients exhibit retarded body growth, hypopigmentation, hypocholesterolemia and low levels of neurotransmitters. Here we report first attempt at creating a homozygous *Pah* knock-out (KO) (Hom) mouse model, which was developed in the C57BL/6 J strain using CRISPR/Cas9 where codon 7 (GAG) in *Pah* gene was changed to a stop codon TAG. We investigated 2 to 6-month-old, male, Hom mice using comprehensive behavioral and biochemical assays, MRI and histopathology. Age and sex-matched heterozygous *Pah*-KO (Het) mice were used as control mice, as they exhibit enough PAH enzyme activity to provide Phe and Tyr levels comparable to the wild-type mice. Overall, our findings demonstrate that 6-month-old, male Hom mice completely lack PAH enzyme, exhibit significantly higher blood and brain Phe levels, lower levels of brain Tyr and neurotransmitters along with lower myelin content and have significant behavioral deficit. These mice exhibit phenotypes that closely resemble PKU patients such as retarded body growth, cutaneous hypopigmentation, and hypocholesterolemia when compared to the age- and sex-matched Het mice. Altogether, biochemical, behavioral, and pathologic features of this novel mouse model suggest that it can be used as a reliable translational tool for PKU preclinical research and drug development.

## Introduction

Phenylketonuria (PKU) is an autosomal recessive inborn error of l-phenylalanine (Phe) metabolism. It is caused by a partial or complete deficiency of the enzyme phenylalanine hydroxylase (PAH) activity, which is necessary for conversion of Phe to tyrosine (Tyr), due to mutations in the *Pah* gene^1–3^. This metabolic error results in buildup of Phe and secondary metabolites and lower Tyr concentration in blood and in brain, which causes degenerative neuropathology and neurological symptoms and intellectual deficits^4–7^. A neonatal screening has been successfully implemented in Europe, USA, UK, Canada, Japan and China to establish diagnosis and carrier identification of PKU^8,9^. Once confirmed, all patients are clinically monitored and require a lifelong treatment to slow the development of neuropathology that is associated with PKU. Treatment is centered on dietary modification to include specially manufactured diet that is low in protein and Phe free. However, maintaining adequate adherence to such low-protein, low Phe diet is challenging. Patients that do not adhere to the diet regimen or that are clinically non-diagnosed exhibit progressive development delay, characteristic poorly or non-pigmented skin and hair, mousy odor, neurological symptoms, cognitive deficits, and behavioral and social problems^10,11^. Lower levels of brain neurotransmitters including dopamine, serotonin, homovanillic acid (HVA), and 4-hydroxyindoleacetic acid (5-HIAA) are frequently observed in PKU^12^.

Among various available mouse and rat models of PKU, *Pah*^enu2^ mice have been extensively used in preclinical assessment which has resulted in better understanding of PKU disease biology. The *Pah*^enu2^ mouse model was generated via nitrosourea-induced chemical mutagenesis in the BTBR strain^13^. It has a F263S missense mutation in the catalytic domain of mouse PAH protein^13,14^ that results in normal transcription of *Pah* mRNA but generation of an inactive PAH protein, which has negative effect on the wild-type protein subunits. The active PAH enzyme works as a dimer and tetramer due to presence of C-terminal oligomerization domain; therefore, incorporation of the mutant full-length protein reduces activity of the enzyme complex. Hence the magnitude of correction when testing therapeutics that rely on production of active PAH enzyme is underestimated^15^. Moreover, degenerative neuropathology characterized by myelin disorder which is often observed in early and continuously treated PKU patients has not been consistently reported in this model^16^. The BTBR mice, background strain for *Pah*^enu2^, exhibit corpus callosum agenesis and hippocampal commissure defect which can impact behavior and assessment of brain pathology endpoints^17,18^. Additionally, use of protein-based assays for assessment of efficacy or confirmation of mechanism of action for biotherapeutics such as *Pah* gene replacement or *Pah* mRNA in this model can be challenging as most of the analytical antibodies cross-react to both mouse and human PAH protein^19^.

To provide an alternative PKU model with no endogenous PAH protein produced, we generated a novel *Pah* knock-out (KO) mouse model by introducing a stop codon at the very beginning of the *Pah* (codon 7) gene. This is the first attempt at creating *Pah*-KO mouse model and as such prevents the production of mouse PAH protein and therefore eliminates any background PAH detection. The lack of mouse PAH protein, such as the F263S protein in the *Pah*^enu2^ model, also prevents the interference of mouse mutant PAH protein with any therapeutic human PAH protein introduced. All patients with the early stop codons and most mutations in the human PAH catalytic domain (i.e. mutations R243X, G261X, and G272X, all in exon 7) are null mutations resulting in a severe PKU^3^. Our mouse model was developed in C57BL/6 J strain using CRISPR/Cas9 where codon 7 (GAG) in *Pah* gene was changed to TAG resulting in conversion of GAG (Glu) to TAG (stop codon). We then investigated *Pah*-KO mouse model using comprehensive behavioral and biochemical assays, MRI and histopathology.

## Results

### Generation of *Pah*-KO mice

**Figure 1:**
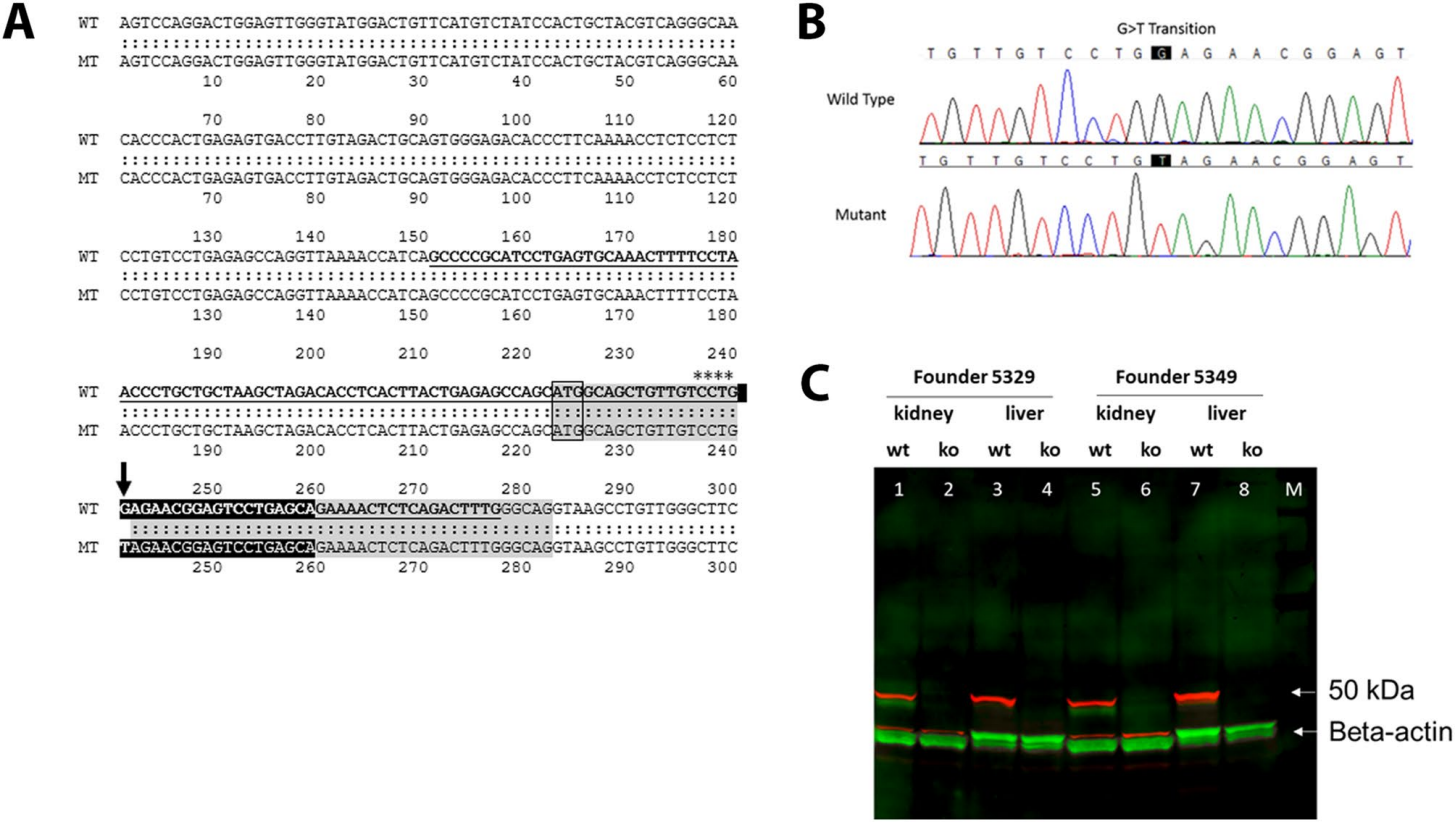
Creation of a *Pah*-KO mouse model. (**A**) The mouse genomic locus on chromosome 10 and the murine targeted allele after CRISPR gene editing are shown. Exon 1 contains the translation initiation codon. NCBI GeneID: 18478; RefSeq transcript: NM_008777. Exon 1 sequence is shadowed gray, the start codon (ATG) is framed, gRNA is shown as white letters on a black background, the asterisks and arrow indicate the position of PAM and G>T transition. The donor oligo for CRISPR targeting is in bold and underlined. (**B**) Sequencing genomic DNA samples of the founder 5349. The point mutation of G to T is indicated in the black boxes. (**C**) Western blot analyses to detect PAH protein in liver or kidney of wildtype (wt) or Hom (ko) mice. Solubilized proteins (60 µg) from mouse liver or kidney were fractionated and immune-blotted with an anti-PAH antibody. The PAH protein (red; expected size 50 kDa) is evident in wildtype mice (lanes 1, 3, 5 and 7) but is absent in Hom mice (2, 4, 6 and 8); lanes 1, 2, 5, 6) are from kidney; lanes 3, 4, 7, 8 are from liver. Equal loading is indicated by probing with antibody to beta-actin (green). M, molecular weight marker lane (bands not visible in this image).

The constitutive KO of *Pah* allele was obtained by inserting a stop codon in exon 1 of the gene via CRISPR/Cas9-mediated gene editing. The affected codon 7 was transitioned from GAG to TAG (Glutamic Acid to Stop), which created a premature transcript (Fig. 1A,B). The Cas9 mRNA along with the gRNA2 (antisense) and single stranded (sense strand) donor oligonucleotides were injected into C57BL/6 J (#000664) zygotes. After recovery approximately 30 micro-injected one-cell stage embryos were transferred to one of the oviducts of 0.5-day post-coitus pseudopregnant CByB6F1/J females. In total 360 embryos were transferred in 4 independent sessions which resulted into birth of 64 pups. Two mutant founders (#5329 and 5349) were further characterized. The genotype status of pups was confirmed by PCR on the genomic DNA extracted from the tail biopsies (Table 1). The *Pah*-KO status was validated by murine *Pah* gene sequencing. The lack of *Pah* expression in livers of Hom mice was confirmed using qRT-PCR (data not shown). No PAH protein was detected in livers or kidneys of Hom mice by Western blot analysis (Fig. 1C). Additional characterization for the two founder lines included analyses for blood Phe levels, liver enzymes and lipid values (Fig. S1). One of the founder lines (#5349) was then selected for expansion into heterozygotes (Het) and homozygotes (Hom) breeding pairs and for production of study cohorts.

**Table 1:** The genotype of breeding animals was identified by genomic tail DNA probed with PCR applying indicated primer pairs and cycling conditions.

Sequence	CATGGCAGCTGTTGTCCTG(**g/t**)AGAACGGAGTCCTGAGCAG	
PCR primers	Fwd	5′-CCTAACCCTGCTGCTAAGCTA-3′
	Rev	5′-AACAGGCTTACCTGCCCAAA-3′
	WT-probe Hex	5′-TTGTCCTG**G**AGAACGGAGTC-3′
	KO-probe 6-FAM	5′-TCCTG**T**AGAACGGAGTCCTGA-3′
PCR product size	WT-Fwd & WT-Rev	WT: 117 bp
	KO-Fwd & WT-Rev	KO: 117 bp
Program	95 °C 3′ 95 °C 5″, 60 °C 30″, 40 cycles	

Taqman qPCR protocol was run on a real time PCR instrument using an appropriate instrument specific Fluorophore/Quencher combination. The transgene genotype is determined by comparing ΔCt values of each unknown sample against known homozygous and heterozygous controls, using appropriate endogenous references.

### Hom mice lack hepatic PAH protein and exhibit higher blood Phe and lower Tyr levels

**Figure 2:**
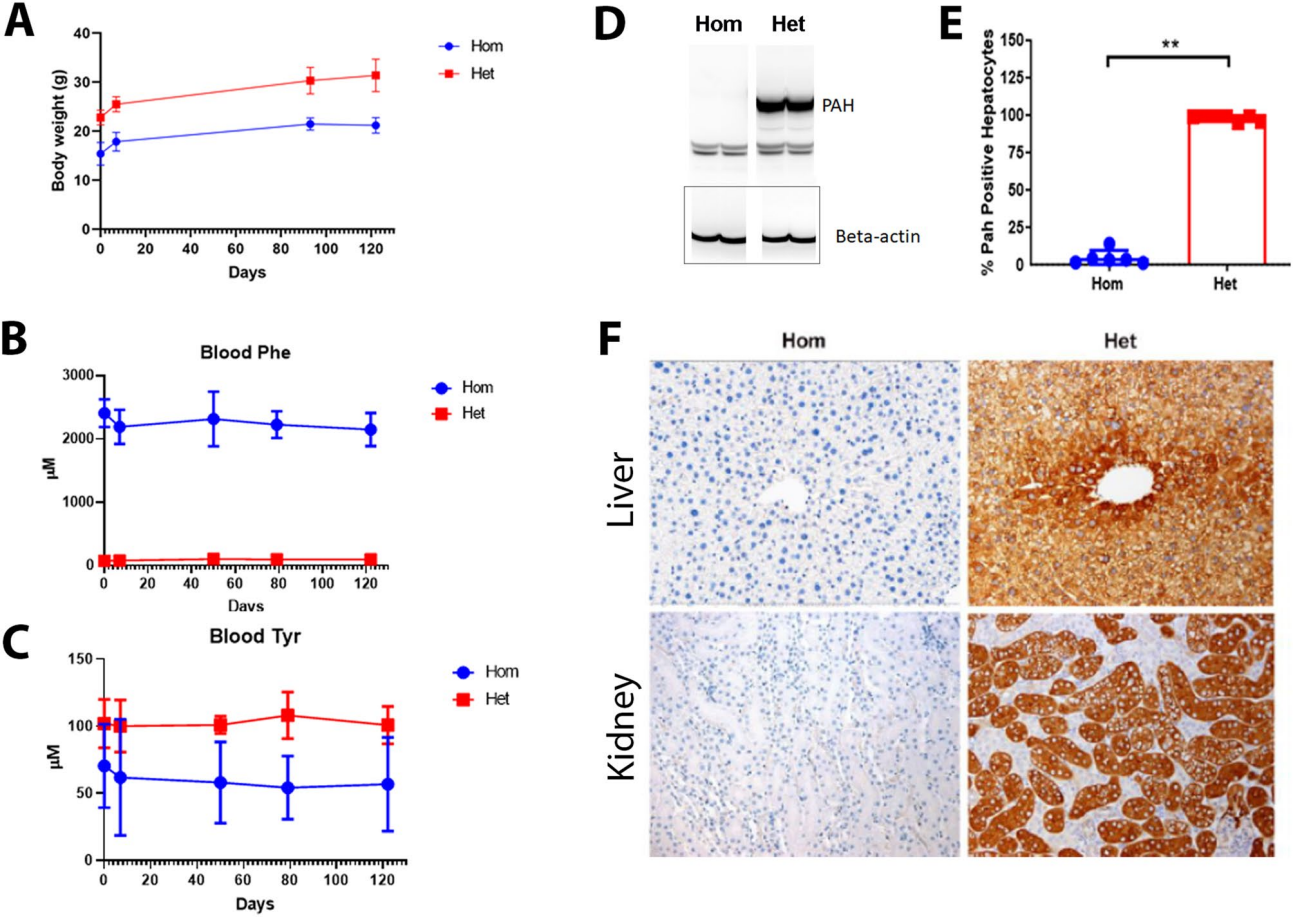
Characterization of Hom and Het *Pah*-KO mice for 6 months. (**A**) The Hom mice showed lower bodyweights than the Het mice throughout the study (**B**) Summary of blood Phe at various timepoints is shown (n = 7/group) (**C**) Summary of blood Tyr at various timepoints is shown (n = 7/group). The graphical data are represented as mean ± SD (Phe and Tyr levels in Hom vs Het mice *p* < 0.0001) (n = 7 mice/group). (**D**) Western blot analysis of liver homogenate using anti-PAH antibody is shown. Note the lack of PAH protein in the Hom mice whereas Het mice showed a corresponding band (~ 50 kDa) for mouse PAH protein. Each lane contained 135 µg protein. Beta actin was used as an internal control. Full-length blots/gels are presented in Supplementary Fig. 2. (**E**) Summary of % PAH positive hepatocytes in the Hom and Het mice are shown. The graphical data are represented as mean ± SD (**p = 0.0012). (**F**) Representative liver and kidney PAH-IHC images show lack of IHC signal in a Hom mouse whereas liver from a Het mouse exhibit strong IHC signal in hepatocytes, mostly in the centrilobular region. Renal cortical epithelial cells from a Hom mouse show no IHC signal as compared to Het mouse, where most proximal convoluted tubular epithelium exhibit strong signal.

Cohorts of Hom and Het mice were characterized up to 6 months of age. During this time, the bodyweights of Hom mice were consistently lower than Het mice (Fig. 2A). Phenylalanine and Tyr levels in blood were measured at 2, 3, 4, 5, and 6 months of age. At all these time points, mean Phe levels in Hom mice were significantly (*p* < 0.0001) higher and Tyr levels were significantly lower (*p* < 0.0001) than in Het mice (Fig. 2B,C). No age-dependent changes for Phe and Tyr levels were observed. After termination of the animals, Western blot analysis showed no hepatic PAH protein at 6-months (Fig. 2D and Fig. S2). The lack of PAH protein expression in liver and kidney was further confirmed by anti-PAH immunohistochemistry (IHC). By digital quantitative image analysis, significantly (*p* = 0.0012) higher percentage of PAH-positive hepatocytes was observed in Het mice than in the Hom mice (Fig. 2E). Phenylalanine hydroxylase-positive signal was present in 98–99% hepatocytes in Het mice in contrast to Hom mice, which showed weak IHC staining that was judged non-specific background signal. Qualitative analysis revealed intense cytoplasmic IHC signal in most hepatocytes of Het mice, whereas such signal was not observed in the hepatocytes of Hom mice (Fig. 2F, top panel). In Het mice, the signal intensity was most prominent within the centrilobular hepatocytes. Similarly, PAH-positive signal was not observed in the kidneys of Hom mice, whereas strong signal was observed in proximal convoluted tubular (PCT) epithelium in Het (Fig. 2F, bottom panel) mice.

### Hypocholesterolemia, and hepatic and renal histopathology in Hom mice

**Figure 3:**
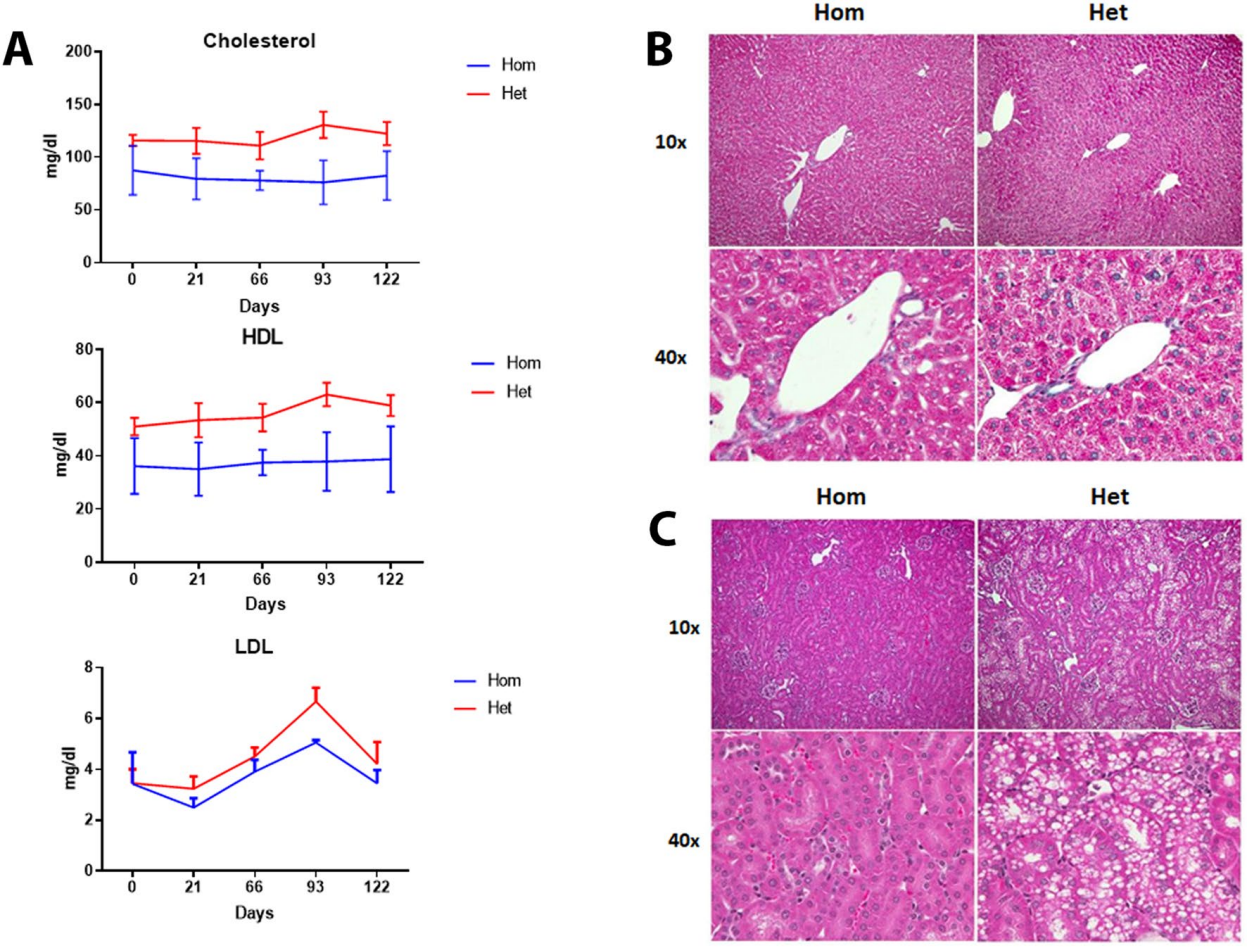
Liver and kidney histopathology and analysis of sera lipid values. (**A**). Analysis of serum total cholesterol, HDL and LDL in Hom and Het mice at various timepoints. The graphical data are represented as mean ± SD. (n = 7/group) (Hom vs Het mice—***p ≤ 0.0008 Cholesterol and HDL levels, *p = 0.02 LDL levels) (n = 7 mice/group). (**B**) Representative liver and H&E-stained images. Corresponding liver histopathology was mostly unremarkable between the two groups except for subtle microvesicular change in the periportal hepatocytes of Hom mice. (**C**) Representative kidney H&E-stained images. Renal images show distinct vacuoles in PCT epithelial cells of a Het mouse while these are absent in Hom mouse.

Serum total cholesterol, HDL and LDL levels were measured at 2, 3.5, 4.5, 5.5 and 6 months of age. Mean cholesterol, HDL, and LDL levels in Hom mice were significantly (*p* < 0.05) lower than in Het mice (Fig. 3A).

For liver histopathology, small numbers of lymphocytes, macrophages and rare plasma cells were present within the perivascular regions, portal triads, or randomly within the hepatic lobules in both Hom and Het mice (Fig. 3B). These changes were observed in approximately 15% Hom (1/7) and Het (1/7) mice and were attributed to the background strain. Hepatocytes either diffusely or within the periportal regions contained cytoplasmic indistinct microvesicles and central nuclei. Approximately 15% of Hom and Het mice exhibited morphological features of congenital portosystemic shunt such as duplication of arteriolar profiles along with the inconspicuous portal veins, dilated lymphatic vessels, and hypertrophic arterial tunics. Portosystemic shunts have been reported previously in C57BL/6 mice^20^. Serum liver enzymes (ALT and AST) were measured and revealed no differences between the two groups.

In the kidney, the proximal convoluted tubular (PCT) epithelial cells contained single to multiple, variably sized (up to 6–8 µm in diameter), discrete vacuoles in up to 90% of Het mice (Fig. 3C). Vacuoles were occasionally displacing and compressing the nucleus into a crescent shape. In 10–20% of the animals, these vacuolated epithelial cells contained multiple or pyknotic nuclei indicative of regeneration and degeneration. Similar vacuoles were not observed in the kidneys of Hom mice.

In kidneys of both Het and Hom mice, tubular proteinaceous casts (minimal severity; 1–2 casts/tubule per kidney section) were observed in 20–30% of the mice. Similarly, small foci of medullary interstitial mineralization of low severity were observed in up to 20–30% of the mice. These renal finding are common background/spontaneous changes of aging mice with none to minimal clinical significance^21^.

### Brains of Hom mice contain higher levels of Phe and lower levels of neurotransmitters and Tyr

**Figure 4:**
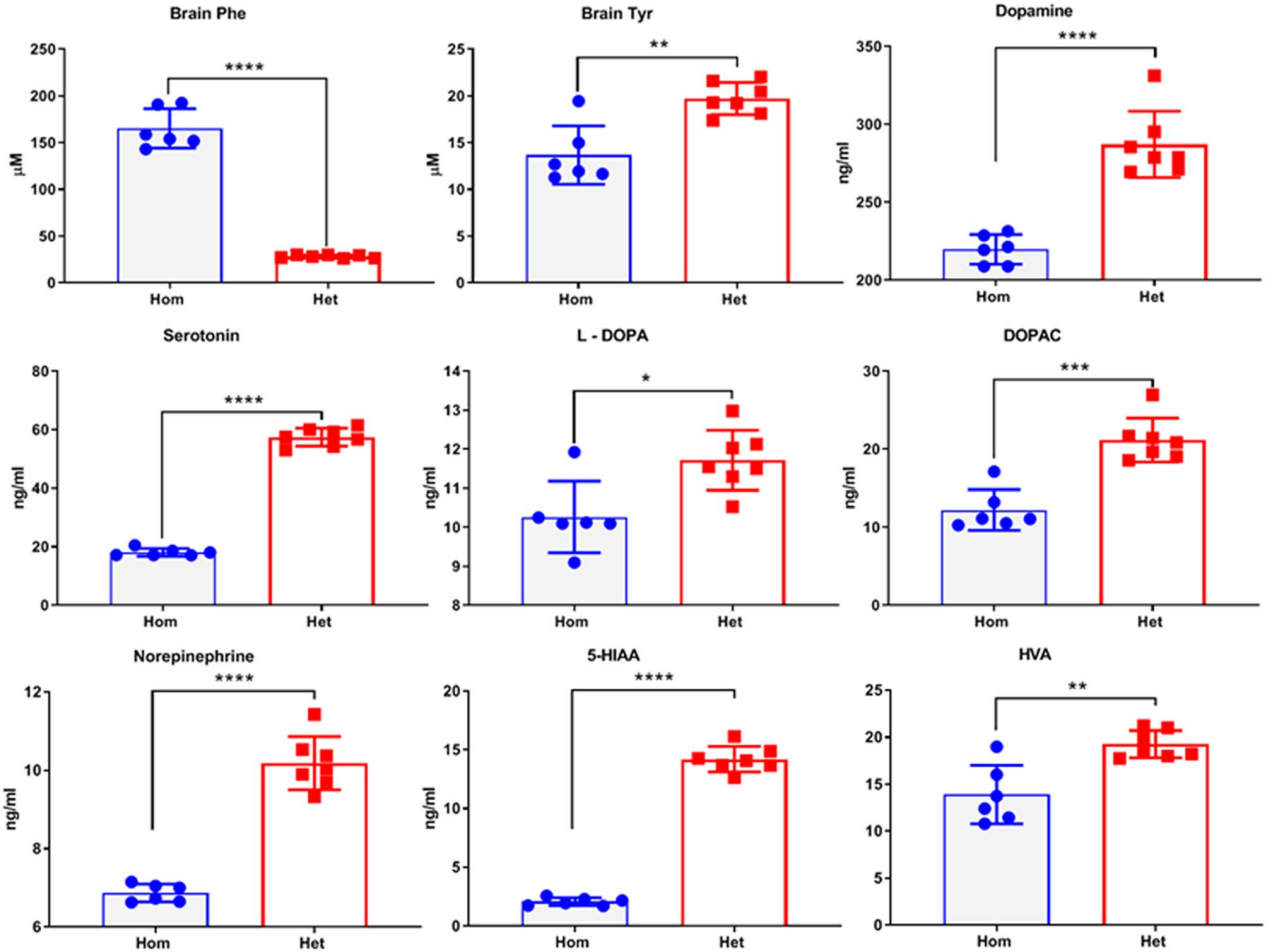
Analysis of Phe, Tyr and neurotransmitters in brains of Hom and Het *Pah*-KO mice at 6 months. Scatter plot charts show brain Phe, Tyr, serotonin, dopamine, L-DOPA, DOPAC, NE, 5-HIAA, and HVA. n = 6 Hom, 7 Het. Bars indicate group mean and the error bars indicate standard deviation (*p = 0.01, **p = 0.003—0.007, ***p = 0.0001, ****p < 0.0001).

Similar to PKU patients, mean Phe content in whole brain homogenates of Hom mice at 6 months was significantly (*p* < 0.0001) elevated when compared to that in age-matched Het mice (165.2 µM in Hom versus 28.2 µM in Het). In contrast to Phe, mean Tyr level in brain of 6-month-old Hom mice was 13.7 µM, whereas these contents were significantly (*p* = 0.0033) higher in brain of Het mice at 19.7 µM (Fig. 4). Neurotransmitters including dopamine, serotonin, norepinephrine, L-DOPA, 5-HIAA, HVA and DOPAC (3, 4-Dihydroxy phenylacetic acid) in brains of Hom mice were significantly (*p* < 0.05) lower than the brain of Het mice (Fig. 4).

### Hom mice display hypomyelination and lower brain and body weight

**Figure 5:**
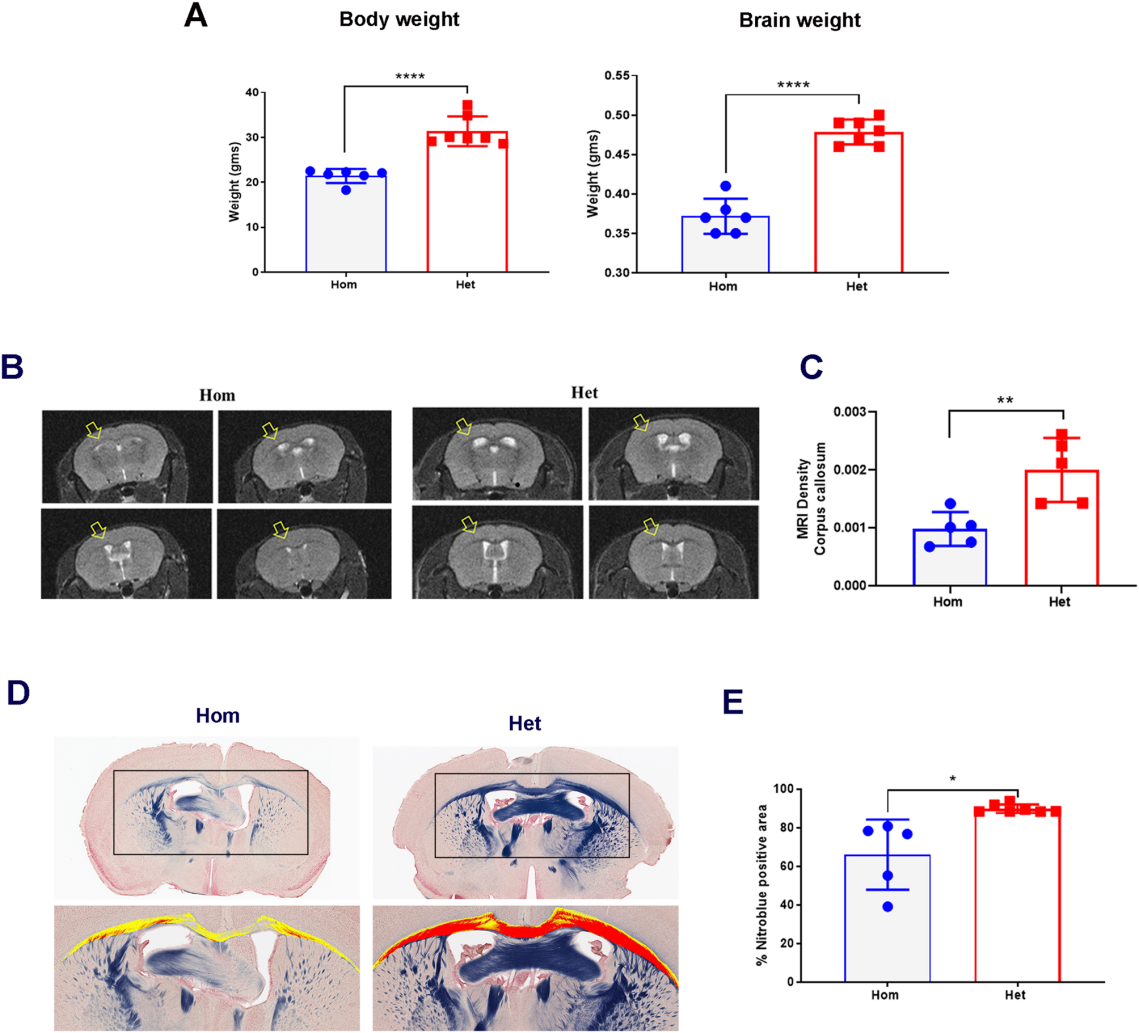
Brain and body weights and brain myelin changes in Hom and Het mice. (**A**) Summary of brain and body weights in 6-month-old Hom and Het mice. n = 6 Hom, 7 Het. (**B**) Representative MRI images showing corpus callosum density between Hom and Het mice. Note extensive distribution of the myelin change in Hom mouse. Each panel represents an individual animal at one time point. (**C**) Scatter plot shows MRI density in corpus callosum. n = 5 Hom, 5 Het. (**D**) Nitroblue stained representative images showing difference in myelin staining intensity in corpus callosum of 6-month-old Hom and Het mice. Note relatively pale blue intensity in the corpus callosum of a Hom mouse as compared to the Het mouse. Yellow (pseudo) color demonstrates sparse myelin content in corpus callosum of a Hom mouse when compared to the intense red representing dense myelin in corpus callosum of a Het mouse. Processed images annotated to include corpus callosum for signal quantitation using HALO2.2% area algorithm. Nitroblue stain. (4×). (**E**) Scatter plot shows % myelin positive area when compared to the Het mice. Nitroblue stained sections analyzed using HALO2.2% area algorithm. n = 6 Hom, 7 Het. The graphical data for (**A,** **C, E**) are represented as mean ± SD (****p < 0.0001, **p = 0.0065, *p = 0.0428).

At 6-months of age, mean body weight of Hom mice (21.42 g ± 1.57) was significantly (*p* < 0.0001) lower than the mean body weight of Het mice (31.37 g ± 3.30) (Fig. 5A). Mean brain weight in Hom mice was 0.36 g ± 0.021, which was significantly (*p* < 0.0001) lower than the Het mice at 0.48 g ± 0.016 (Fig. 5A). Brain myelin contents were assessed using *in-vivo* MRI on whole brains and nitroblue histochemical staining of fixed coronal brain sections (Fig. 5B,D). T2-MRI was performed at 6-months of age and revealed significantly (*p* = 0.01) lower brain myelin density (MRI signal ratio) at 3.68 ± 0.14% in the Hom mice when compared to the myelin density (MRI signal ratio) of 5.36 ± 0.37% in Het mice (Fig. 5C). Similar differences were observed in brain and corpus callosum volume (data not shown). For myelin quantitation in nitroblue stained sections, whole slides were scanned, and myelin was digitally quantified in manually annotated images that included corpus callosum. Nitroblue positive area was quantified using area algorithm (HALO2.2) and revealed significantly lower nitroblue positive area (myelin) in corpus callosum of Hom mice when compared to the Het mice (66.0% nitroblue positive area in Hom vs 89.8% in Het mice; *p* = 0.04) (Fig. 5E). Qualitatively, the corpus callosum region in Hom mice appeared to have reduced cell density (decreased number of glial cells that are morphologically suggestive of oligodendrocytes), compared to the Het mice.

### Ophthalmic pathology in Hom mice

**Figure 6:**
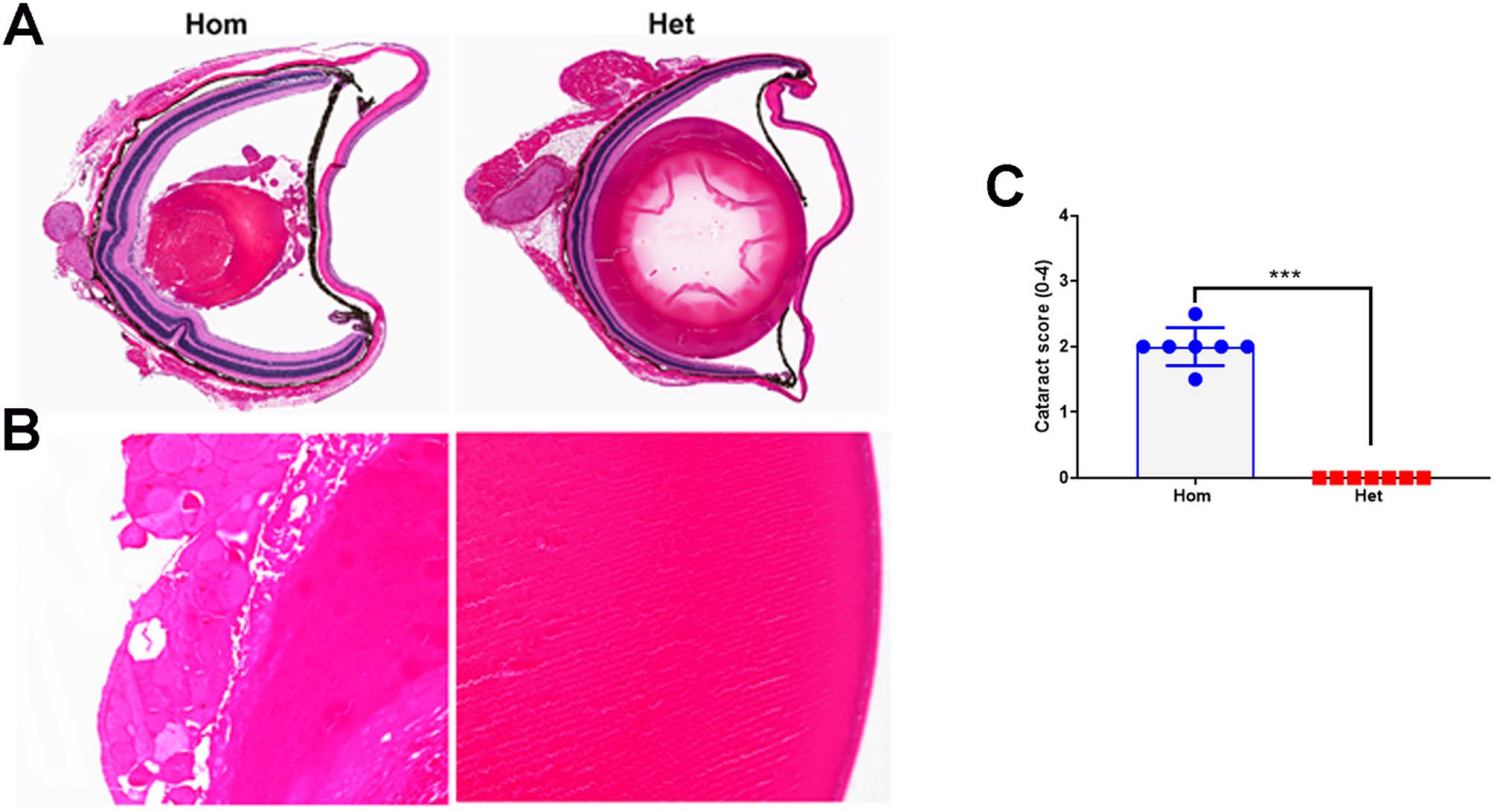
Cataract in Hom mice. (**A**) Note hypermature cataract characterized by collapsed and fragmented lens with the wrinkled capsule and formation of amorphous non-hyalinized core surrounded by protein globules in a Hom mouse as compared to the Het mouse. H&E stain. 1×. Higher magnification of lens showing disorganized, fragmented, and swollen lens fibers forming Morgagnian globules and hypereosinophilic condensed core in Hom mouse (**B**). H&E stain. 20×. (**C**) Summary of cataract score is shown in the scatter plot graph. n = 7 Hom, 7 Het. Bars indicate group median score and the error bars indicate range (***p = 0.0006).

Hom mice exhibited variable degrees of ophthalmic pathology. At 6-months of age, right microphthalmia was observed in 6/7 Hom mice whereas a similar change was not observed in Het mice. However, in the follow-up study to investigate this change at earlier time points such as 2.5 months and 4.5 months, microphthalmia (Davidson’s fixed specimens) was observed in both eyes in Hom mice but with the predilection for the right eye. Microscopically, cataract characterized by bladder cell and Morgagni globule formation involving either the peripheral or complete lens was the prominent lesion in all Hom mice at 2.5–6 months of age (Fig. 6A,B). In 2 mice, the lens capsule was collapsed, wrinkled or ruptured and the lens protein was extruded within the vitreous humor. In one mouse, rare bands of fibrous tissues were observed between collapsed lens and iris. The extent of cataract was assessed using a semiquantitative scheme (grade 0—within normal limit; grade 1—cataract involving less than 50% lens; grade 2—cataract involving more than 50% lens with our without membrane wrinkles; grade 3—cataract with lens rupture and grade 4—collapsed intraocular layers). Median grade of ocular pathology at 6 months was significantly (*p* = 0.0006) higher in Hom mice when compared to the Het mice (Fig. 6C). Other associated histologic changes included posterior synechiae in 2 of 7 Hom mice, and corneal edema, ulceration and neovascularization in 1 of 7 Hom mice.

### Hom mice exhibit hypopigmented hair coat

**Figure 7:**
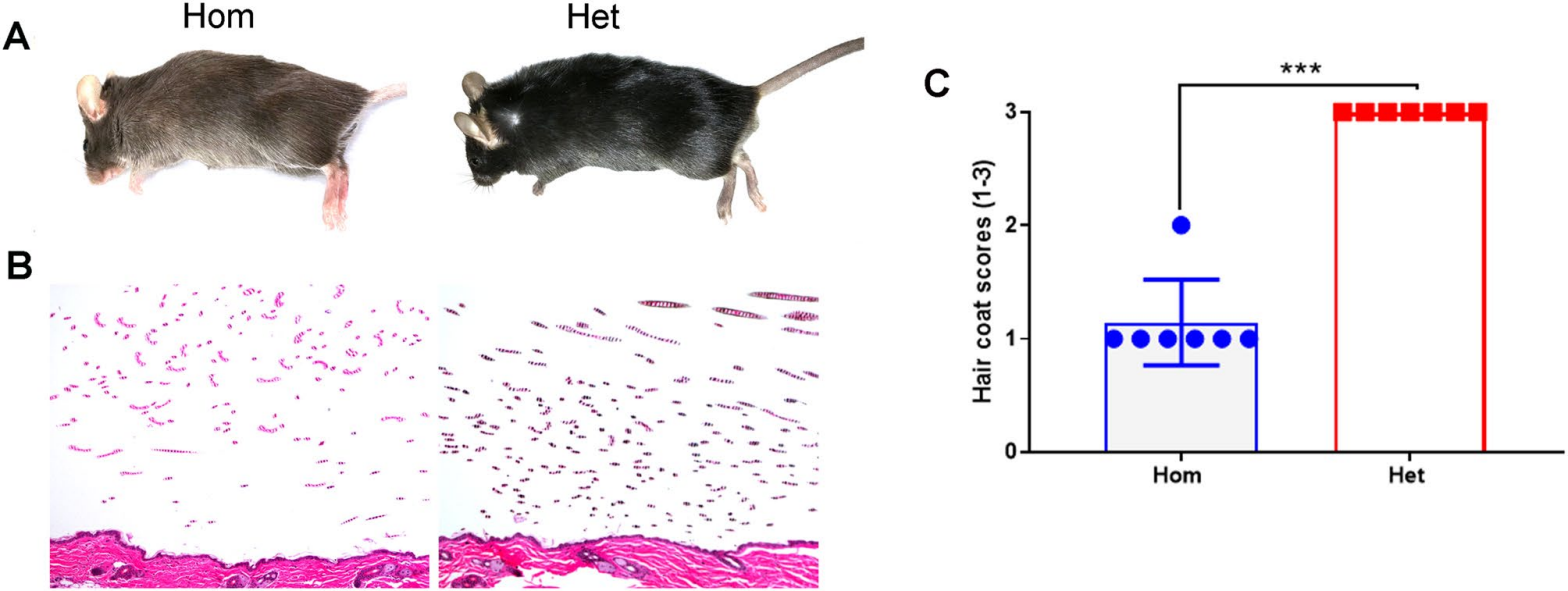
Hair coat color in Hom-*Pah* KO mice. The difference in hair coat color is grossly visible (**A**). Hair coat of the Hom mouse is light brown whereas it is dark brown for the Het mouse. (**B**) Hair shafts from the Hom mouse contain less melanin pigment when compared to the Het mouse. H&E stain 20× (**C**) Summary of hair melanin scores is shown in the scatter plot graph. n = 7 Hom, 7 Het. Bars indicate group median score and the error bars indicate range (***p = 0.0006).

Hair coat of 6/7 (85.71%) Hom mice was light brown whereas 1/7 (14.28%) was brown. In contrast, all 7/7 Het mice had dark brown hair coat color at 6 months (Fig. 7A). Histologically, hair shafts contained variable amount of melanin pigment (Fig. 7B) which was assessed using a semi-quantitative grading scheme (score 1—poorly pigmented hair shafts, score 2—moderately pigmented hair shafts and score 3—well pigmented hair shafts). Median score for the presence of melanin in hair shafts of Hom mice was significantly (*p* = 0.0006) lower when compared to the Het mice (Fig. 7C).

### Hom mice display progressive behavioral deficit

**Figure 8:**
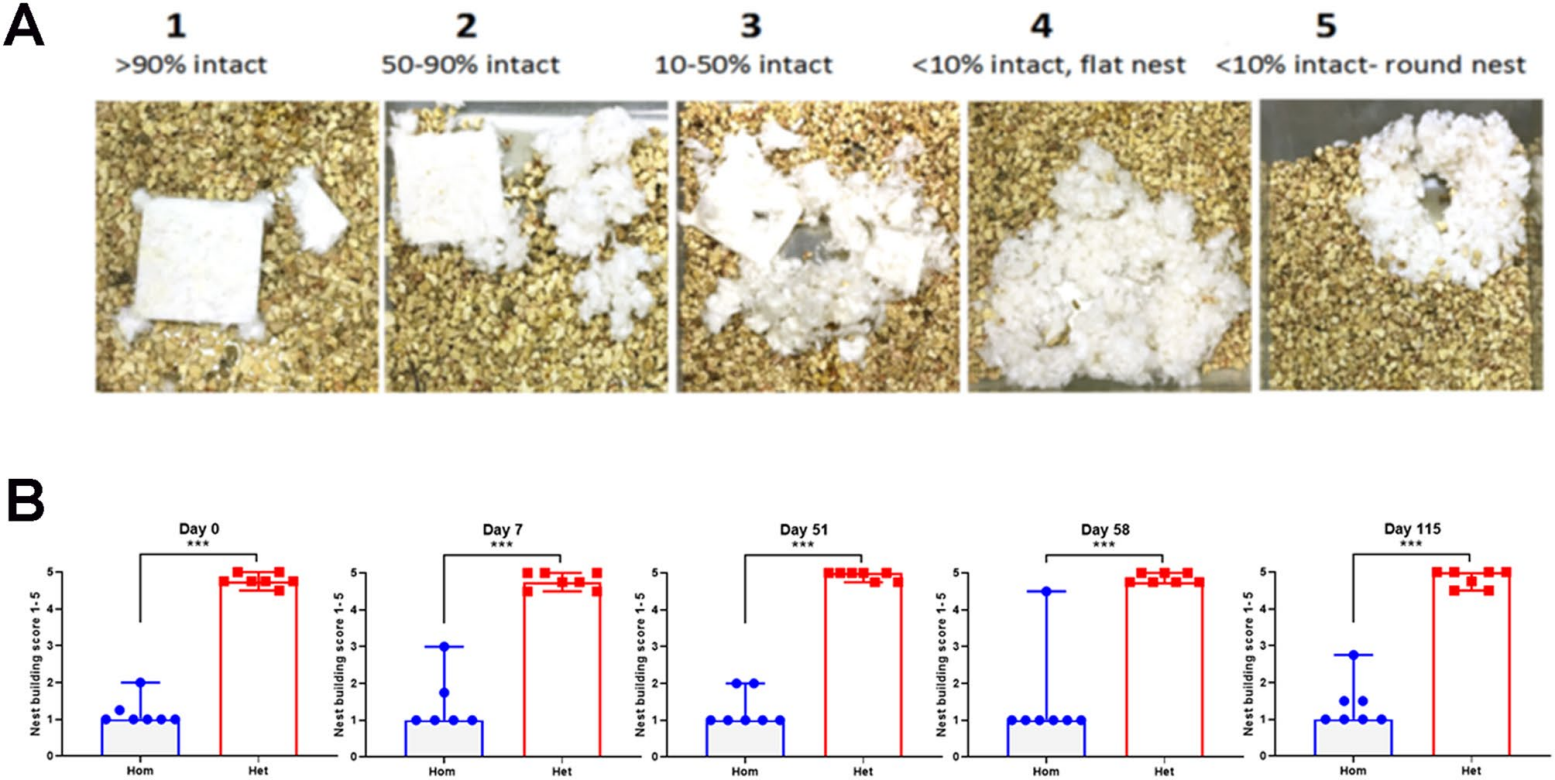
Nest building assay for behavioral competence. (**A**) Nesting behavior was assessed in the home cages using packed cotton material. The mice first shred the tightly packed material, then arrange it into a nest overnight. Semiquantiative score, shown above the images, is assigned based on the quality of the nest. (**B**) Scatter plot graphs show individual animal nesting score. n = 7 Hom, 7 Het. Group columns represent median scores and the error bars indicate range in Hom and Het mice at various timepoints (***p = 0.0006).

Nest building assay to assess the behavioral competency was performed at 2, 2.5, 4, 4.5 and 6-months of age (Fig. 8A,B). Nesting behavior was performed in the home cages of mice that were provided with pressed cotton material. In this assay, the mice first shred the tightly packed material, then arranged it into a nest overnight^22^. Score was then assigned based on the quality of the nest and weighing any unused material. The latter allowed fine-tuning the quality scores and overall correlated well with the qualitative nest scoring data (Fig. S3). For the hom mice, the group median scores at all time points ranged from 1.18 to 1.5 (individual animal range from 1 to 4.5). In Het mice, group median scores at all time points ranged from 4.78 to 4.93 (individual animal range from 4.5 to 5). Median group scores at all time points in Hom mice were significantly (*p* = 0.0006) lower when compared to the Het mice (Fig. 8B).

## Discussion

Here we report creation of a homozygous (Hom) *Pah* knock-out (KO) mouse model for preclinical assessment of PKU. Our mouse model was developed in the C57BL/6 J strain using CRISPR/Cas9 where G in codon 7 of *Pah* gene was changed to T resulting in conversion of GAG, Glu to TAG which is a stop codon. We then investigated 2 to 6-month-old, male, Hom mice using comprehensive behavioral and biochemical assays and histopathology. Age and sex-matched Het mice were used as the control group as they have normal plasma Phe levels. The Hom mice were generated via targeted knockout of the *Pah* gene and lacked *Pah* mRNA expression and PAH protein in liver. *Pah*-KO status was additionally confirmed by gene sequencing. Absence of PAH protein was confirmed using western blot analysis of liver and kidney homogenate, which was further corroborated by anti-PAH immunohistochemistry. When compared with the Het mice, the Hom mice exhibited significantly lower PAH protein in liver and kidney using immunohistochemistry. Furthermore, no liver PAH activity was detected using assay measuring Tyr production (data not shown). Blood Phe levels at 2, 3, 4, 5 and 6 months of age were consistently higher, and brain Phe levels at 6-months were also significantly higher in the Hom mice as compared to that of age-matched Het mice. Similar to PKU patients, Hom mice exhibited retarded body growth. PKU represents a heterogeneous group of disorders which is reflected in the underlying genetic diversity. A large number of mutations in the *Pah* gene have been reported^3^; some cause complete absence of PAH enzyme activity whereas others result in residual activity of PAH enzyme. In the most severe form (classic PKU), the patients have a mutation that results in complete absence of PAH activity. In this regard our mouse model resembles the classic form of PKU where the Phe levels are higher than 1200 μmol/L.

Despite extensive biochemical characterization of PKU disease biology and positive correlation between hyperphenylalaninemia and altered neurological, behavioral and cognitive deficits, the pathophysiology of this disability remains poorly understood. It has been suggested that competitive transport inhibition of neutral amino acids such as histidine, tyrosine, tryptophan, threonine, valine, methionine, isoleucine and leucine at the blood brain barrier by high Phe levels inhibits protein and myelin synthesis^5,7^. Additionally, Phe as an essential amino acid is crucial for the synthesis of Tyr and its catecholamine derivative neurotransmitters such as dopamine, norepinephrine, and epinephrine. PKU patients show reduced levels of brain dopamine, serotonin, NE, HVA, and 5-HIAA and tyrosine^7, 12,23^. It has been proposed that lower levels of Tyr adversely affect dopaminergic neurons in prefrontal cortex resulting in cognitive deficits^24^. The Hom mice in our study exhibited significantly higher blood and brain Phe and lower Tyr levels as compared to the Het mice when assessed at various time points. Similar to PKU patients, brain levels of dopamine, serotonin, NE, HVA, and 5-HIAA were significantly lower in Hom mice. Oxidative stress in PKU brain pathology has also been proposed^25^. It has been hypothesized that oxidative stress secondary to an increased plasma Phe level would cause alterations in brain energy metabolism and trigger the generation of reactive species^25,26^. In that regard, we performed inducible nitric oxide synthase (iNOS) IHC on brain sections but did not observe significant differences between Hom and Het mice (data not shown).

Neuropathology characterized by brain white matter abnormalities is often observed in PKU patients. There is a positive correlation between the severity of white matter change and the degree and duration of hyperphenylalaninemia^4^. Untreated PKU children show variable white matter abnormalities ranging from hypomyelination, reduced oligodendrocyte numbers, status spongiosus and frank demyelination^23^. These changes are regionally specific, such as status spongiosus is prominent in the optic tracts, periventricular white matter, centrum semiovale, and cerebellar white matter. Myelin changes, which are observed in patients with prolonged hyperphenylalaninemia have predilection for corpus callosum and the periventricular white matter. The Hom-*Pah*^*enu2*^ mice have been used to address this aspect of PKU biology; however, hypomyelination and demyelination have not been consistently observed in this model. In the Hom mice, we detected distinct brain white matter changes which included hypomyelination in corpus callosum. This was demonstrated by magnetic resonance imaging and nitroblue biochemical stain at 6 months of age. Nitroblue positive corpus callosum and brain density ratio, assessed using MRI, were significantly lower in the Hom mice when compared to age and sex-matched Het mice. Myelin water imaging by MRI has been shown to correlate well with histological myelin staining^27^.

Altered lipid profile characterized by significantly lower blood total cholesterol, LDL and HDL has been observed in some PKU patients^28–31^. It is not clear whether these differences are due to the low protein diet or disruption of cholesterol biosynthesis. However, recent findings suggest that hyperphenylalaninemia rather than the low protein diet is the cause of altered lipid profile in PKU^30,31^. The underlying mechanism for low cholesterol biosynthesis in PKU patients is also not clear but it is widely believed that Phe induced inhibition of 3-hydroxy-3-methylglutaryl-CoA reductase (HMG-CoA), the rate limiting enzyme for cholesterol synthesis could be the cause of low cholesterol. In fact, experimentally induced hyperphenylalaninemia in chickens and *Pah*^enu2^ mice has been shown to inhibit the brain and/or liver HMG-CoA activity^32,33^. Similar to those with PKU, low serum total cholesterol and low HDL were observed in the Hom mice at 2, 3.5, 4.5, 5.5 and 6 months of age. Interestingly, all animals received regular 16% protein diet which further supports previous finding that hyperphenylalaninemia rather than diet could be the cause of hypocholesterolemia. Additionally, cholesterol is an indispensable component of myelin membranes and its availability in oligodendrocytes is essential for adequate myelination. Saher et al.^34^ demonstrated that the mutant mice which are incapable of cholesterol synthesis have myelin defect. It will be interesting to see if there is reduced cholesterol synthesis in the brain of Hom mice and if there is an association between impaired myelination and low cholesterol.

Ocular abnormalities have been described in PKU patients and include blindness, albinoid fundus, blue irises, cataract, and microphthalmia^35–37^. Other than albinoid fundus and blue irises, a direct link between PKU and cataract has been contradictory. Cotticelli et al.^37^ reported congenital cataract in 1/14 PKU patients. Zwaan^36^ reported cataracts in 6/11 PKU patients and concluded that eye abnormalities except hypopigmentation occurred due to self-trauma or treatment with high doses of psychotropic agent thioridazine hydrochloride. Histopathological changes in the eyes of Hom mice were dominated by bilateral cataract in various stages of progression, leading to the lens rupture and secondary uveitis of phacoclastic nature in some Hom mice. To understand these ocular changes, we further investigated ophthalmic pathology at 2.5 and 4.5 months of age in Hom mice. Interestingly bilateral cataract was consistently observed, and the severity of cataract histopathology correlated positively with Phe levels in some animals. Presence of bilateral cataract in our model and the rat offsprings of maternal PKU model suggest a possible link between PKU and cataract^38^. Right microphthalmia was observed in several Hom mice and some Het mice; this change is a background C57BL/6 J strain related trait^39,40^ and was considered unrelated to Pah deficiency in the Hom mice.

One of the major clinical phenotypes in PKU patients is hypopigmentation. Patients have fair skin, blond hair, and blue eyes, while their unaffected parents and siblings have pigmented skin, hair and brown eyes^41^. This hypopigmentation has been attributed to lack of melanin production. Melanogenesis is a multistep process that begins with hydroxylation of Tyr to DOPA by tyrosinase. DOPA is subsequently converted to dopaquinone which then gives rise to melanin. Lower Tyr, a substrate for tyrosinase, and competitive inhibition of Tyr uptake in melanocytes by Phe contribute to hypopigmentation in PKU^42^. As expected, blood Tyr levels at 2, 4, 5 and 6 months of age were significantly lower in the Hom mice as compared to the higher Tyr levels in Het mice. Indeed, individual animal hair coat color, when assessed macroscopically, correlated well with the blood Tyr level. Hom mice with low blood Tyr levels had light brown hair coat whereas Het mice with normal blood Tyr levels had dark brown shade of hair coat. In contrast to humans where melanin is present in epidermis and hair shafts, melanin in mice is restricted to the hair shafts. Therefore, Hom mice with the light brown hair coat had significantly lower degree of melanin pigmentation in hair shafts, when assessed using histopathology, as compared to the darker shade. Differences in melanin granule color and size were not visible between the Hom and Het mice. Interestingly, a Hom mouse which had dark brown hair coat contained blood Tyr level that was comparable to the Het mice. Presence of this clinical correlate in our model increases extrapolation value and, when applicable, suggests assessment of cutaneous pigment restoration as a clinical endpoint. We also assessed melanin pigment in retina using semiquantitative grading scheme but did not observe similar differences between the two groups (data not shown). It is possible that retinal melanin developmental pathways are different in humans and mice.

To assess the impact of the biochemical changes on behavioral phenotype, we performed nest building assay where the nesting score is assigned based on the quality of the nest and the weight of any unused material. Assessing both these aspects allows robust detection of behavioral deficit and has been used for validating species-typical behavior^43,44^. The nest building assay has been reported to measure changes in anxiety, depression and motor defects. Thought the exact biochemical basis for the changes in the nest building behavior is unclear, it has been reported to correlate well with reduction in brain neurotransmitter levels, cholesterol synthesis and white matter content^44,45^. In our study, the nest scores and unused nesting material values were significantly lower for the Hom mice at all timepoints.

In conclusion, we generated *Pah*-KO mice on the C57BL/6 background using CRISPR/Cas9 where codon 7 (GAG) in *Pah* gene was changed to a stop codon (TAG). Our findings demonstrate that the Hom *Pah*-KO mice recapitulate PKU disease biology, metabolic perturbation, and biochemical and clinical phenotypes. The *Pah-*KO mouse model has no detectable PAH in liver or kidney and demonstrates consistent brain hypomyelination, lower tyrosine and neurotransmitters and behavioral deficit. Hence, this model can be used to study PKU biology resembling classic PKU patients. It is also ideal for assessing the efficacy of various modalities of liver-targeted *Pah* gene replacement therapies, delivery of *Pah* mRNA and gene editing techniques such as CRISPR/Cas9 or TALENS, where understanding the level of liver correction via PAH detection is important. Hence, our model offers an alternative preclinical PKU model that should be helpful for assessment of various new therapeutic strategies for the treatment of PKU.

## Methods

### Animals

All animal experiments were performed in an animal facility accredited by the Association for Assessment and Accreditation of Laboratory Animal Care International (AAALAC) in compliance with the ARRIVE guidelines (http://www.nc3rs.org.uk/page.asp?id=1357) and relevant regulations using animal use protocols approved by the Sanofi Institute Animal Care and Use Committee. Mice were kept and bred under specific and opportunistic pathogen-free conditions (SOPF) in the animal care facility. A colony of *Pah*-KO mice was maintained at Jackson Laboratories. Breeding strategy involved HET × HET mating. Homozygous and heterozygous male mice were obtained at approximately 2 months of age and were housed in accordance with humane guidelines for animal care and use. Animals were genotyped for the absence of gene at weaning using custom designed TaqMan assay (Table 1) on tail biopsy samples at Jackson. Additionally, the phenotype was confirmed by measuring blood Phe levels. During the 6-month characterization study, all animals were housed at individual cages and received regular 16% protein diet. For termination, animals were anesthetized with isoflurane, followed by blood collection from retro-orbital plexus into EDTA or sera collection tubes. Animals were then humanely euthanized by CO2 asphyxiation and subjected to intracardiac perfusion with PBS. Various tissue samples were collected and either frozen at − 80 °C until analysis or fixed in 10% NBF for histopathology.

### Generation of *Pah*-KO mice

The constitutive KO of *Pah* allele was obtained by inserting a stop codon in exon 1 of the gene via CRISPR/Cas9-mediated gene editing. The affected codon 7 was transitioned from GAG to TAG (Glutamic Acid to Stop), which created a premature transcript (Fig. 1A). The guide RNA (antisense gRNA2, TGCTCAGGACTCCGTTCTCC) and the donor oligo (sense donor1, GCCCCGCATCCTGAGTGCAAACTTTTCCTAACCCTGCTGCTAAGCTAGACACCTCACTTACTGAGAGCCAGCATGGCAGCTGTTGTCCTGTAGAACGGAGTCCTGAGCAGAAAACTCTCAGACTTTG, the mutated nucleotide is marked by bold font and the CRISPR PAM sequence is underlined) were designed and checked for their specificity and low incidence of off-target binding (Fig. 1A and Table S1) using Benchling algorithm (https://benchling.com) which provided off-target scores based on the Doench et al., guide^46^. The donor oligo did not have any silent mutations in it, as the desired SNP disrupted the PAM. The Cas9 mRNA along with the gRNA2 and single stranded (sense strand) donor were injected into C57BL/6 J (#000664) zygotes. After recovery approximately 30 micro-injected one-cell stage embryos were transferred to one of the oviducts of 0.5 dpc, pseudopregnant CByB6F1/J females. In total 360 embryos were transferred in four independent sessions which resulted into birth of 64 pups. PCR-screening and sequence validation confirmed seven females and six males positive for the desired mutation. The mutant founders 5329 and 5349 were further characterized and the founder 5349 was selected for expansion and production of study cohorts (hetero- and homozygotes). The *Pah*-KO status was further identified and validated by confirming G>T transition in exon 1 of the murine *Pah* gene using Sanger sequencing (Fig. 1B) and lack of *Pah* protein using Western blots (Fig. 1C). The genotypes of pups were confirmed by PCR on the genomic DNA extracted from the tail biopsies. The PCR conditions along with the used primers, probes, and the fragment sizes are provided in the Table 1. Briefly, Taqman qPCR protocol was run on a real time PCR instrument using an appropriate instrument specific Fluorophore/Quencher combination. The transgene genotype was determined by comparing ΔCt values of each unknown sample against known homozygous and hemizygous controls, using appropriate endogenous references.

### Phe and Tyr level analyses

The plasma Phe and Tyr levels were analyzed by UHPLC-MS/MS, using Transcend II LX4 multiplex system equipped with Dionex Ultimate 3000 HPLC (Thermo Fisher, Waltham, MA, USA) hyphenated to an API 4000 triple quadrupole mass spectrometer (AB SCIEX, Framingham, MA, USA). L-Phe and L-Tyr (Sigma-Aldrich, St. Louis, MO USA) were used to prepare standard solutions, and labeled L-Phe-^13^C_9_, ^15^N and labeled L-Tyr-^13^C_9_, ^15^N (Sigma-Aldrich) were used as internal standards. The MS/MS detection was carried out in positive ion mode. The analysis was performed using an Acquity BEH C18 column (1.7 μm, 2.1 × 30 mm) with gradient separation, which included a 10 s hold at 98% mobile phase A (0.1% formic acid in water) followed by a 2–45% mobile phase B (0.1% formic acid in acetonitrile) gradients over 30 s, an increase to 75% B over 15 s, a 10 s wash at 75% B, and re-equilibration at 98% A for 1 min at a 0.5 mL/min flow rate. MS/MS transitions were: 166.1/120.1 for Phe, 182.1/136.1 for Tyr, 176.1/129.1 for labeled Phe, and 192.1/145.1 for labeled Tyr. Brain Phe and Tyr levels were quantitated similarly.

### Serum cholesterol

Total serum cholesterol, high-density lipoproteins (HDL) and low-density lipoproteins (LDL) were measured on Daytona analyzer using enzymatic endpoint method (Randox, RX Series CH 3810, Crumlin, United Kingdom).

### Liver and kidney PAH protein analysis

Phenylalanine hydroxylase protein was detected in liver and kidney specimens using Western blot. Briefly, the samples were homogenized twice in tubes with ceramic beads (15-340-153; ThermoFisher Scientific 168 Third Ave Waltham MA 02451) in 50 mM KPO_4_, pH 7.0, 1% KCl, 1 mM DTT with 1 × protease inhibitors at 5.65 m/s at 4 °C for 20 s in homogenizer (Omni Bead Ruptor-24). Samples were spun at 21,000*g* at 4 °C for 45 min. Supernatants were then analyzed by Western blot as described previously with minor modifications^47^. Briefly, mouse monoclonal anti-PAH (LSBio, LSC344145) was used as primary antibody and donkey anti-mouse IgG-HRP (LSBio LS-C60841) as secondary antibody. The signal was detected using Femto Signal Western ECL substrate (ThermoFisher Scientific, 168 Third Ave, Waltham MA-02451). The PAH enzyme activity in liver homogenates was measured as previously described with some minor modifications^48^. Briefly, the assay mixture contained liver homogenates in 100 mM Tris–HCl, pH 7.5, 4 mM DTT, 5 mM l-phenylalanine and 2300 U bovine catalase in a volume of 250 μL. The reaction was initiated by the adding DMPH4 to a final concentration of 0.5 mM and was carried out at 24 °C for 30 min with shaking.

### Neurotransmitter analyses

Neurotransmitters were measured in brain samples. Brains were processed as described with minor modifications^49^. Briefly, half of sagittal brain sections from PBS perfused animals were weighed and ice-cold lysis buffer (1 mM oxalic acid, 3 mM cysteine, 0.1 M acetic acid) was added at 200 mg/mL wet weight. Samples were then homogenized using Bead Ruptor 24 at 4.85 m/s for 20 s at 4 °C. The homogenized samples were centrifuged at 14,000 RPM for 10 min. Supernatant was collected and frozen at − 80 °C and later used for quantifying l-Dopa, Dopamine, Homovanillic acid (HVA), Serotonin, 5-Hydroxytryptophan (5-HTP), 5-Hydroxyindoleacetic acid (5-HIAA) and Norepinephrine levels by UPLC-MS/MS, using an Acquity UPLC (Waters Corporation, Milford, MA, USA) hyphenated to an API 5000 triple quadrupole mass spectrometer (AB SCIEX, Framingham, MA, USA). To increase the stability of neurotransmitters, 300 ng/mL Cysteine in 0.1% formic acid (FA) and acetonitrile was used as the sample diluent buffer. Standards for each analyte (Sigma-Aldrich) and standard solutions were prepared similar to the samples as described above. The MS/MS detection was carried out in positive ion mode for all neurotransmitters except HVA. The analysis was performed using an Acquity for HSS C18 SB (1.7 μm, 2.1 × 100 mm) with gradient separation, which included a 0.5 min hold at 98% mobile phase A (0.1% FA in water) followed by a 2–40% mobile phase B (0.1% FA in acetonitrile) gradient over 3.5 min, an increase to 95% B over 0.1 min, a 0.5 min wash at 95% B, and re-equilibration at 98% A for 2.4 min at a 0.5 mL/min flow rate. The positive ion MS/MS transitions were: 198/152 for l-Dopa, 177/160.1 for Serotonin, 154/137.1 for Dopamine, 192/146 for 5-HIAA, 170.1/107 for Norepinephrine, 221.1/201 for 5-HTP. Homovanillic acid detection was carried out in negative ion mode, similar to the method described above except that the initial hold phase of the gradient was eliminated. The MS/MS transition for HVA in negative ion mode was 181.1/137.1. The levels were expressed as μg/mL homogenate or μM.

### In vivo MRI assessment

In vivo mouse brain MRI was performed using a Bruker Biospec 70/30 horizontal bore system (Bruker, MA, USA) equipped with 460 mT/m gradients and a 36-mm diameter volume radiofrequency coil (Animal Imaging Research, Holden, MA, USA)^50^. Multi-slice T2-weighted (T2W) coronal images were acquired using a RARE sequence (TR/TE = 2500/33 ms, 15 averages, 0.5-mm thickness, matrix size = 175 × 175, FOV = 18 mm^2^). For volumetric and T2W MRI signal intensity analysis, an ROI was drawn around the brain and visible corpus callosum structure across the coronal slices to calculate the corpus callosum volume and the average brain and corpus callosum signal intensities. The quantification of MRI signals and volumetric analysis were done by using AMIRA (Thermo Scientific, MA, USA) and Cheshire (PAREXEL, MA, USA) software.

### Histopathology and immunohistochemistry

Tissues were collected and fixed in 10% neutral buffered formalin. Tissues were then routinely processed, embedded in paraffin, sectioned at 5 µm, and stained with hematoxylin and eosin (H&E). Coronal brain sections were trimmed on Vibratome (50 µm) and stained with nitroblue as described previously utilizing 10% neutral buffered formalin fixative^51^. Slides were scanned (Aperio, ScanScope XT system) and images were annotated to include corpus callosum. Percentage nitroblue positive signal in corpus callosum was quantified using HALO2.2 percentage area algorithm.

Formalin fixed paraffin embedded liver, kidney and brain were processed by automated immunohistochemistry staining for anti-PAH (BOSTER, A00761-1) or anti-inducible nitric oxide synthase (iNOS) (abcam, ab15323) antibody using the BondRX autostainer (Leica Biosystems) following a standard protocol for rabbit antibodies. Briefly, the sections (previously baked at 60 °C for 30 min) were deparaffinized with Dewax Solution (Leica, AR9222) then subjected to an antigen retrieval process with sodium citrate pH 6.0 (Leica, AR9961) for 20 min at 100 °C followed by peroxide block (Bond Polymer Refine DAB Detection kit, Leica Biosystems, DS9800) then serum free protein block (DAKO X0909). Primary antibodies anti-PAH or anti-iNOS were diluted to 1 µg/ml or 0.13 µg/ml, respectively, in antibody diluent, (DAKO, S3022) and incubated for 30 min at room temperature. Anti-rabbit polymer (Leica, Refine kit, DS9800) was used as a secondary antibody for 20 min at room temperature. The slides were then subjected to DAB (Leica Biosystems, DS9800) for 5 min, hematoxylin (Leica Biosystems, DS9800) for 10 min and bluing solution (Richard-Allan Scientific, 7301) for 1 min before rinsing in DI water, drying at 60 °C for one h and applying coverslip.

### Nest building behavior assay

A published protocol^22^ was used with minor modifications. Briefly, each animal was moved into a clean, individual cage and a 3.0 g ± 0.02 square of cotton (Nestlet; cabfm00088, Ancare) was placed into each cage. The next day, any unused bedding material was weighed and the quality of nest was scored based on the following rating scale of (1) Nestlet not touched (more than 90% intact), (2) Nestlet partially torn (50–90% intact), (3) Nestlet mostly shredded but often without identifiable nest site (less than 50% intact), (4) An identifiable but flat nest (more than 90% of the Nestlet is torn), (5) perfect nest with a crater and high walls (more than 90% of the Nestlet is torn) (Fig. 5). Scoring was performed by two individuals and the average score was used.

### Statistical analysis

Statistical comparisons were made using GraphPad Prism 7.02 (GraphPad Software, La Jolla, CA, USA). For parameters that were assessed using semi-quantitative grading schemes, results were analyzed using one-way ANOVA and nonparametric Kruskal–Wallis and Dunn’s multiple comparisons tests. Quantitative parameters were evaluated using parametric Unpaired t tests with Welch’s correction. *p* < 0.05 was considered to indicate statistically significant differences.

## Supplementary Information

Supplementary Information.
