# Supplementary material for: CRISPR/Cas9 generated knockout mice lacking phenylalanine hydroxylase protein as a novel preclinical model for human phenylketonuria
Source: Sci Rep. 2021 Mar 31;11:7254. doi: 10.1038/s41598-021-86663-8 (PMC8012645; doi:10.1038/s41598-021-86663-8)

**SUPPLEMENTARY MATERIAL**

**CRISPR/Cas9 generated knockout mice lacking phenylalanine hydroxylase protein as a novel preclinical model for human phenylketonuria**

Kuldeep Singh^1,7^, Cathleen S. Cornell^2^, Robert Jackson^2^, Mostafa Kabiri^3a^, Michael Phipps^3^, Mitul Desai^4^, Robert Fogle^4^, Xiaoyou Ying^4^, Gulbenk Anarat-Cappillino^5^, Sarah Geller^5^, Jennifer Johnson^1^, Errin Roberts^1^, Katie Malley^1^, Tim Devlin^3^, Matthew DeRiso^3^, Patricia Berthelette^2^, Yao V. Zhang^2,8^, Sue Ryan^1^, Srinivas Rao^6^, Beth L. Thurberg^1^, Dinesh S. Bangari^1*^ , Sirkka Kyostio-Moore^2*^

^1^Global Discovery Pathology, Translational In-Vivo Models Research Platform, Sanofi, 5 The Mountain Road, Framingham, MA-01701, United States

^2^Genomic Medicine Unit, Sanofi, 49 New York Avenue, Framingham, MA-01701, United States

^3^Transgenic Model and Technology, Translational In-Vivo Models Research Platform, Sanofi, 5 The Mountain Road, Framingham, MA-01701, United States and ^3a^Transgenic Model and Technology, Translational In-vivo Research Platform, Industrie Park Hoechst, Sanofi, Frankfurt, Germany

^4^Global Bioimaging, Translational In-Vivo Models Research Platform, Sanofi,

^5^Pre-Development Sciences NA, Analytical R&D, Sanofi, Framingham, MA-01701, United States

^6^Translational In-Vivo Models Research Platform, Sanofi, 49 New York Avenue, Framingham, MA-01701, United States

^7^Present address: WuXi AppTec Inc. 8^th^ Floor, 55 Cambridge Parkway, Cambridge, MA 02142, United States

^8^Present address: 77 Massachusetts Avenue, Cambridge, MA-02139, United States

*Correspondence and requests for material should be addressed to D.S.B (dinesh.bangari@sanofi.com) or S.K.M (Sirkka.Kyostio-Moore@sanofi.com)

**Table S1.** Off-target analysis and scoring of the used guide RNA (gRNA2, antisense). Potential off-target sites, the PAM sequences and their scores are presented.

| **Sequence** | **PAM** | **Score** | **Gene** | **Locus** |
| --- | --- | --- | --- | --- |
| TGCTCAGGACTCCGTTCTCC | AGG | 100 | *Pah* (ENSMUSG00000020051) | chr10:-87522037 |
| TGATAAGGCCTCCGTTCTCC | CGG | 1,6 |  | chr5:+117665156 |
| TGGTCAGCACTCTGTTCTCC | GGG | 1,2 |  | chr2:+61551331 |
| GCCTCACAACTCCGTTCTCC | CAG | 1 |  | chr2:+168911299 |
| TCTTAATGACTCCGTTCTCC | AGG | 1 |  | chr8:-15192764 |

**Supplemental Figures**

**Figure S1.** Analysis of two founder lines (5329 and 5349). (A) Hom male and female mice from both lines had elevated blood Phe levels with females showing higher Phe levels than males. (B) Liver enzymes in sera trended higher in Hom mice compared to Het mice. ALT data in males is shown as an example. (C) Sera lipid values were lower in Hom mice compared to Het mice. HDL values in males are shown as an example. For each analysis, n=2-3 male or female mice were used.


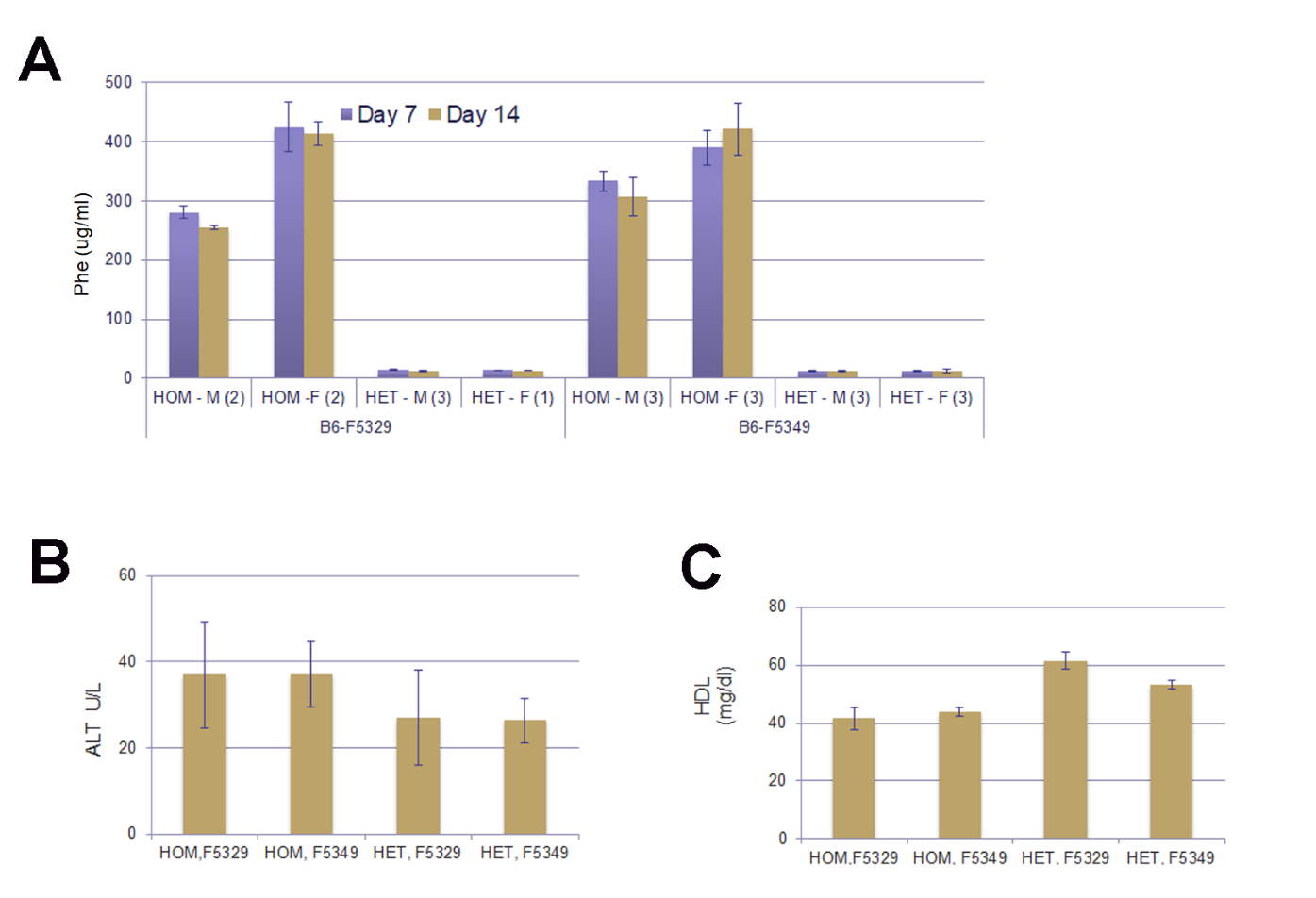


**Figure S2.** Original gel for Fig 2D from which 2 Hom and 2 Het mice are shown for PAH protein detection in livers by Western blot. For each lane 135 ug total protein (determined by BCA assay) were loaded. The PAH Western blot was performed as described in Methods and the signal for PAH was captured. For beta-actin detection (to confirm equal loading for each lane), the membrane was rinsed briefly with PBS.  Membrane was then stripped of PAH antibody by incubation in Restore Western Stripping Buffer (Thermo Scientific 21059) at 37°C for 15 min and then washed twice for 5 min with PBS. Membrane was blocked as described in Methods and incubated with beta-Actin (13e5) Rabbit mAb-HRP conjugated (Cell Signaling #5125S) at 1:5000 dilution for one h. The signal detection was performed as described for PAH. The lanes in beta-actin image were aligned with that of PAH image to visualize PAH and beta-actin levels in all samples. The lanes for Hom (animals 26 and 27) and Het (animals 42 and 43) samples were then cropped from the combined PAH and b-actin image (indicated as blue boxed areas). The grouping of non-contiguous Hom and Het lanes is indicated using a white spacing between the Hom and Het lanes.


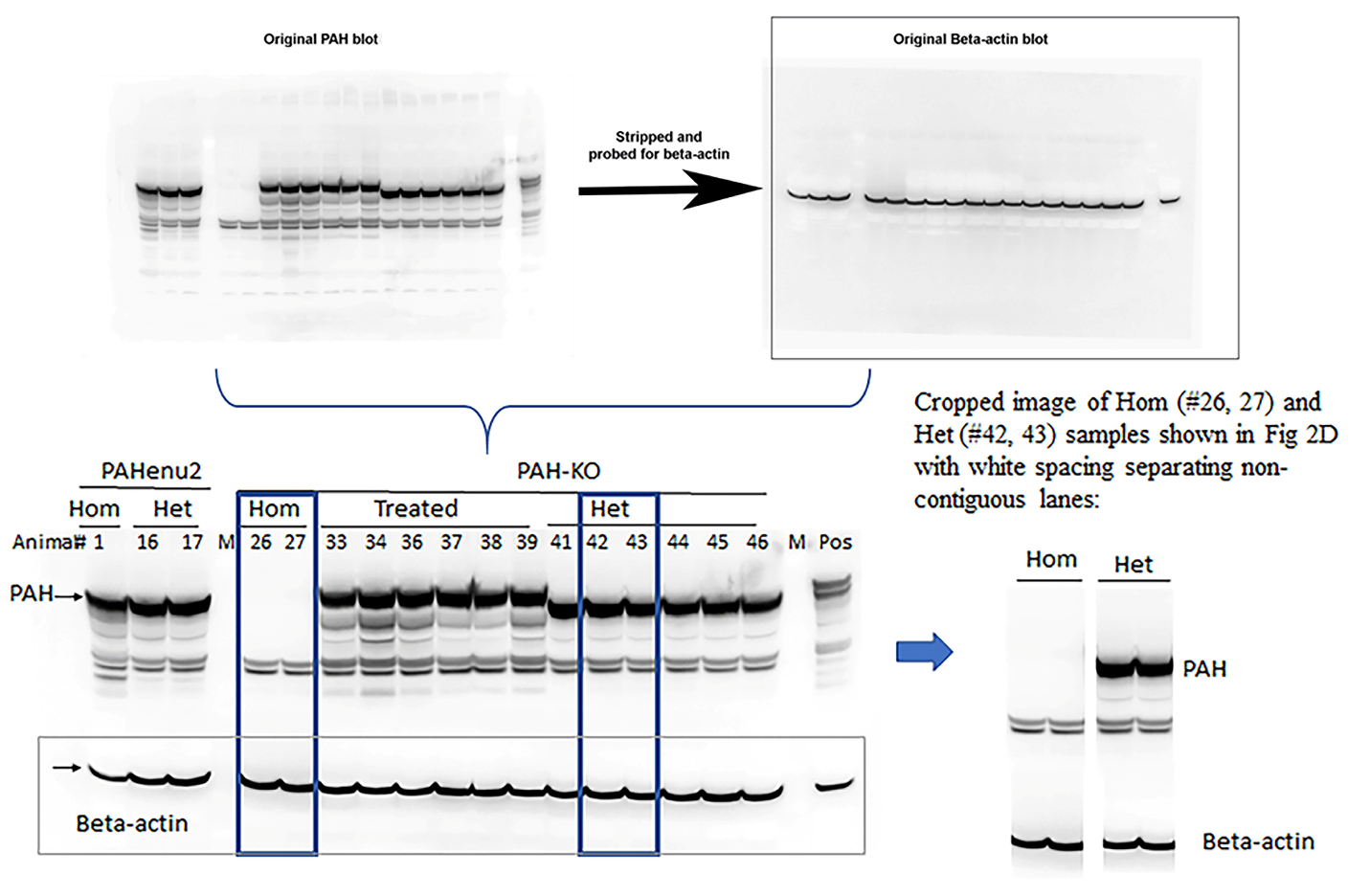


**Figure S3.** Analysis of nesting behavior. (A) Individual animal data for each timepoint is shown. Included are % used nestlet material and scores based of quality of the nest. As an example, (B) the nest score, (C) % used nestlet material and (D) correlation between the two endpoints for Hom and Het mice on day 115 are shown. Note that one Hom animal (#4) is consistently an outlier in all timepoints.


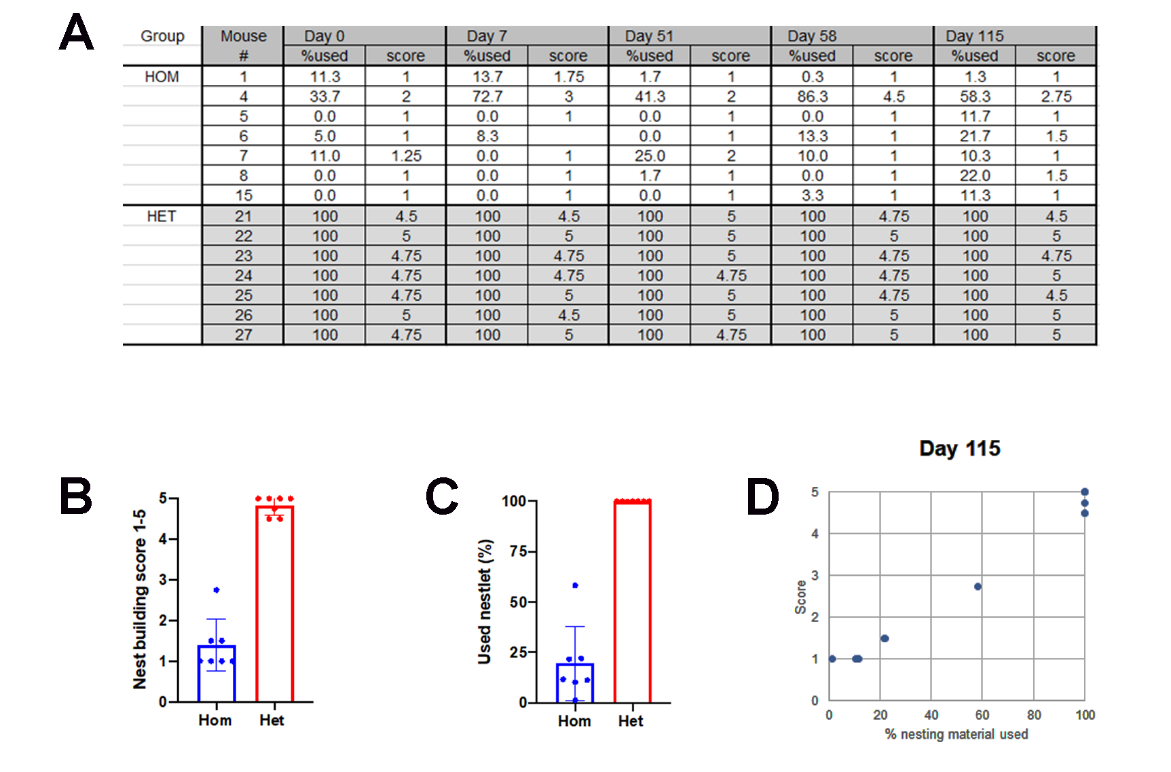

Supplement: Supplementary file 1 — Supplementary Information. [file 41598_2021_86663_MOESM1_ESM.docx]
